# A Black Sticky Rice-Derived Functional Ingredient Improves Anxiety, Depression, and Stress Perception in Adult Volunteers

**DOI:** 10.3390/foods13233884

**Published:** 2024-12-01

**Authors:** Pattamaporn Natthee, Jintanaporn Wattanathorn, Wipawee Thukham-mee, Pongsatorn Paholpak, Poonsri Rangseekajee, Nawanant Piyavhatkul, Suphayanakorn Wattanathorn, Supaporn Muchimapura, Terdthai Tong-Un

**Affiliations:** 1 Department of Physiology and Graduate School (Neuroscience Program), Faculty of Medicine, Khon Kaen University, Khon Kaen 40002, Thailand; pattamapornpoukpralab@gmail.com; 2 Department of Physiology, Faculty of Medicine, Khon Kaen University, Khon Kaen 40002, Thailand; meewep@gmail.com (W.T.-m.); supmuc@kku.ac.th (S.M.); terdthai@kku.ac.th (T.T.-U.); 3 Research Institute for High Human Performance and Health Promotion, Khon Kaen University, Khon Kaen 40002, Thailand; suphawa@kku.ac.th; 4 Department Psychiatry, Faculty of Medicine, Khon Kaen University, Khon Kaen 40002, Thailand; ppaholpak@kku.ac.th (P.P.); poonsri.ran@bkkthon.ac.th (P.R.); nawanant@kku.ac.th (N.P.); 5 Faculty of Medicine, Bangkok Thonburi University, Bangkok 10170, Thailand

**Keywords:** black sticky rice, anthocyanin, dietary inflammatory index, antioxidant quality score, anxiety, depression

## Abstract

We hypothesized that consumption of a diet containing the functional ingredient from black sticky rice, which is rich in anthocyanin, over a five-day period would improve anxiety, depression, and stress perception in adult volunteers based on the benefits of this compound. In this study, a total of 46 male and female adult volunteers with mild and moderate stress level were assigned to groups consuming a breakfast meal containing an anthocyanin-enriched functional ingredient at doses of 2 and 4 g per day for 5 days. The volunteers consumed three meals with a low DII but high DAQ-S, and the total calories consumed during the study period was 2000 kcal/day. Mental well-being, including depression, anxiety, and stress, together with AChE, MAO, Nrf2, 8OHdG, MDA, and the density of *Lactobacillus* and *Bifidobacterium* spp., were assessed at baseline and at the end of the study. Safety parameters were also examined. A diet containing both doses of the anthocyanin-enriched functional ingredient with a low DII but high DAQ-S was found to improve anxiety, depression, and stress, with changes in 8-OHdG and IL-6 levels. No other changes and toxicity-related parameters were observed. Our results show that the novel functional ingredient can improve anxiety, depression, and stress perception partly by decreasing oxidative stress and inflammation; however, randomized, double-blind, placebo-controlled studies with a larger sample size should be performed to confirm this benefit.

## 1. Introduction

At present, mental disorders, particularly anxiety and depression, are regarded as a significant global health burden across the entire lifespan [1]. According to a report by the World Health Organization (WHO), depression is the single highest contributor to global disability, with anxiety ranking sixth in this regard [2]. It has been estimated that the loss of productivity caused by anxiety and depression has a negative impact on the global economy of around USD 1 trillion per year, and this number is predicted to reach USD 16 trillion by 2030 [3]. Around 70% of patients do not receive appropriate and effective care. Serious challenges include relapse, recurrence, and therapeutic resistance [4]. In addition, the results of a study on the cost and efficacy of treatment suggest that there is economic value in preventing these mental disorders [5].

The results of recent studies demonstrate that the pathophysiological processes of depression and anxiety are associated with oxidative stress, which in turn increases inflammation and induces neurotransmitter imbalance and brain plasticity disturbance [4,6]. This raises the possibility that a reduction in inflammation and an increase in antioxidant activity can protect and attenuate both conditions [7,8,9]. Anthocyanins have revealed a positive modulation effect on anxiety and depression [10,11]. In Thailand, many plants such as black-colored rice are rich in anthocyanins [12] and exhibit antioxidant and anti-inflammatory effects [13]. Black sticky rice or *Oryza sativa* L. (var Khao Kum), a special rice cultivar, has been widely consumed in the northeastern region of Thailand since ancient times. This type of rice has a hard texture compared to white sticky rice; therefore, it is not popular among consumers. However, it is believed to provide health benefits due to its high anthocyanin content. Many fruit-derived anthocyanin-enriched substances that contain cyanidin-3-glucoside and cyanidin-3-rutinoside as the main ingredients demonstrate anxiolytic and antidepressant effects [13,14]. The positive modulation effect of the aforementioned fruits containing cyanidin-3-glucoside and cyanidin-3-rutinoside on mental well-being raises the possibility that black sticky rice, which contains both the aforementioned substances and peonidin [15], could serve as a functional ingredient that enhances mental well-being. In addition, our preliminary in vitro data (unpublished data) show that the black sticky rice extract can suppress monoamine oxidase (MAO), which in turn increases the proportion of available monoamine transmitters, including norepinephrine, serotonin, and dopamine, neurotransmitters that play crucial roles in mood disorders [16], including anxiety and depression [17]. Moreover, black sticky rice also possesses antioxidant and anti-inflammatory effects, which can increase the levels of monoamine transmitters, resulting in an improvement in depression and anxiety [4,18]. Based on the high demand for functional ingredients [19] and the high content of anthocyanins in black sticky rice, together with the positive modulation effects on the improvement in neurotransmitter balance, oxidative stress status, and inflammation mentioned above, the development of functional ingredients derived from black sticky rice that aim to enhance mental well-being may be a potential method to increase the value of black sticky rice, which is, at present, not favored among consumers. At present, no clinical study has demonstrated a positive modulation effect of black rice-derived functional ingredients on anxiety, depression, and stress perception in adult volunteers. In addition, most functional ingredients are only applied in the functional food and beverage industry, with no application of these functional ingredients in foods consumed daily. The application of functional ingredients in these foods could expand the market share of functional ingredients to other customer markets, such as restaurants, hotels, and household users. Despite the high demand and significant potential of functional ingredients, no supporting clinical evidence is available at present, which is essential for health benefit claims. Therefore, in this study, we aimed to determine the effect of 5-day consumption of food containing a black rice-derived functional ingredient on anxiety, depression, and stress perception in adult volunteers. The possible underlying mechanisms and safety factors were also explored. Since anthocyanins, the main ingredient in black sticky rice, are poorly absorbed [20], their increased consumption could exert some beneficial effects via the modulation effect on gut microbiota, which leads to the stimulation of the gut–brain axis, leading to an improvement in neuropsychiatric conditions. Changes in microbiota, particularly alteration in the density of *Lactobacillus* and *Bifidobacterium* spp. [21,22], were also investigated.

## 2. Materials and Methods

This study was a randomized, double-blind study. All study protocols were approved by the Center for Ethics in Human Research, Khon Kaen University (HE661401). The study was also registered in the system of the Thai Clinical Trial Registry (TCTR20230811010) and was performed in accordance with the International Conference of Harmonization (ICH) for Good Clinical Practice (GCP) and in compliance with the Declaration of Helsinki and its further amendments. The study was performed at the Faculty of Medicine, Khon Kaen University. Each subject provided written informed consent prior to any trial-related procedures. The Research Institute for High Human Performance and Health Promotion was involved in food preparation throughout the 5-day consumption period.

### 2.1. Diet Preparation

Anthocyanin-enriched powder derived from black sticky rice harvested from Udonthani province, Thailand, in 2023 (cyanidin-3-glucoside 1.116 ± 0.014 mg/g; the fingerprint chromatogram is provided in Supplementary S1), was used as the functional ingredient in this study. Functional food for breakfast was prepared at doses of 2 and 4 g by mixing this functional ingredient with other ingredients. These doses were selected based on our previous data, which demonstrated that anthocyanin-enriched substances at this dosage provide health benefits for brain areas that play a crucial role in executive function, including mood regulation [23]. The consumption of breakfast containing this ingredient throughout the 5-day study period had less potential to induce inflammation and was more likely to exert antioxidant effects. To score inflammatory induction potential, a dietary inflammatory index (DII) or numeric score was used to assess the number of inflammatory foods in the person’s diet. The DII was determined by subtracting the global mean intake from the patients’ reported daily intake and dividing it by the global standard deviation (SD). The obtained result or Z-score was converted to percentiles and multiplied by the corresponding inflammatory effect score. Then, we summed the DII of ingredients to be the DII of the formulation [24,25]. A negative score indicates a low potential to induce inflammation, whereas a positive value indicates proinflammatory potential [26]. The antioxidant potential of food containing this ingredient was assessed using the dietary antioxidant quality score (DAQS) [27]. The DAQS was calculated by comparing the daily nutrient intake of vitamins and minerals with antioxidant effects, such as selenium, zinc, vitamin A, vitamin C, and vitamin E, in the participants compared to the recommended daily intake (RDI). When the intake of five components decreased below 2/3 of the RDI, a value of 0 was applied as well, and a score of 1 was given when the intake exceeded 2/3 of the RDI. Then, the DAQS of each ingredient was summed, yielding the DAQS of the formulation [24]. The DII values of the food containing the functional ingredient from black sticky rice were −2.32, −2.62, −3.71, −2.79, and −3.19; in comparison, the DAQSs were 2.32, 2.62, 3.71, 2.79, and 2.198, respectively. All subjects were required to consume three meals of around 2000 calories per day for 5 days. Detailed information on the breakfast meal used in this study is provided in Supplementary S2.

### 2.2. Study Population and Design

Male and female adult volunteers between the ages of 20 and 60 years were assessed for eligibility and recruited if they met the following inclusion criteria: healthy enough to perform a 6-min walk test; non-smoker; sedentary work and lifestyle; a stress level of no more than moderate or a Sauan Prung Stress Test Questionnaire (SPST-20) score of ≤41; and the ability to communicate in the official language fluently. The exclusion criteria were as follows: having severe physical diseases/conditions that required continuous medication, including heart and blood vessel diseases, respiratory system diseases, nervous system diseases, diabetes, hyperlipidemia, high blood pressure, kidney disease, liver disease, lung or allergic diseases, immune system diseases, blood system diseases, cancer, thyroid disease, adrenal glands, pituitary glands, gout or high uric acid, head injuries, and psychic conditions; taking cholesterol-lowering drugs, blood sugar-lowering drugs, blood pressure-lowering drugs, or drugs that interfere with platelet function; taking other dietary supplements; smoking more than 10 cigarettes per day; consuming more than 5 alcoholic beverages per day; and participating in other projects.

**Figure 1:**
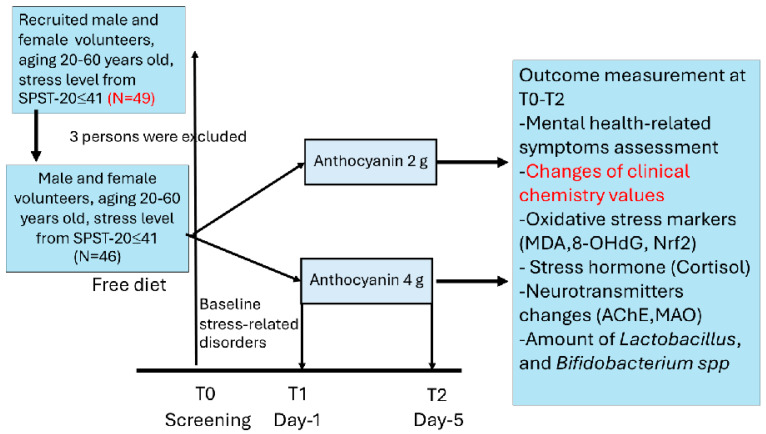
Schematic diagram illustrating the study protocol.

Following screening, 3 individuals were excluded due to dyslipidemia. However, new participants were recruited until the desired number was reached (N = 23/arm). The participants were divided into 2 separate groups: those receiving (1) 2 g of anthocyanins and (2) 4 g of anthocyanins per day. Subjects with mild stress levels were provided with food containing the anthocyanin-enriched functional ingredient at a dose of 2 g/day; in comparison, subjects with moderate stress levels were provided with food containing the anthocyanin-enriched functional ingredient at a dose of 4 g/day. During the 5-day study period, all subjects consumed 3 meals per day. The total calorie intake per day did not exceed 2000 kcal with a low DII and a high DAQ-S. Mental well-being and the suppression of acetylcholinesterase (AChE) and monoamine oxidase (MAO), as well as the levels of cortisol, nuclear factor erythroid 2-related factor 2 (Nrf2), oxidative stress markers such as malondialdehyde (MDA) and 8-hydroxyguanosine (8-OHdG) in serum, and the amount of *Lactobacillus* and *Bifidobacterium* spp. in the participants’ feces, were assessed at baseline and at the end of the study. Details of the study protocol are provided in Figure 1.

### 2.3. Mental Well-Being Assessment

Mental health symptoms were assessed by using the Depression Anxiety Stress Scale-21 (DASS-21)—Thai version [28,29]. This test is a self-report evaluation form consisting of 21 items for evaluating anxiety, depression, and stress. Each domain was evaluated using 7 items and was graded on a 4-point Likert scale ranging from 0 (“did not apply to me at all”) to 3 (“applied to me a lot”). It was found that Cronbach’s alpha coefficients of depression, anxiety, and stress for the Thai version of the scale were 0.82, 0.78, and 0.69, respectively. High scores and high responses reflected the high level of an evaluated condition.

### 2.4. Blood Sampling and Biochemical Assays

Venous blood samples were collected from the volunteers after 12 hours of fasting and used as serum. Blood was prespared as serum and used for the assays. Then, the health-related parameters reflecting safety and toxicity conditions were measured, along with oxidative stress and cellular senescence markers.

Oxidative stress and cellular senescence markers such as MDA (ab287797, Cambridge, England), 8-OHdG (ab285254, Cambridge, England), and Nrf2 (ab277397, Cambridge, England) in serum were assessed using commercially available colorimetric assay kits according to the manufacturer’s protocols.

Safety parameters, including hematological changes, and clinical chemistry values, including serum cortisol levels, were measured at the Clinical Laboratory Unit, Srinagarind Hospital, Faculty of Medicine, Khon Kaen University.

### 2.5. Assessment of Neurotransmitter Changes

We determined the alterations in acetylcholine (ACh) and monoamine transmitters indirectly by measuring the suppression activity of acetylcholinesterase (AChE) and monoamine oxidase (MAO). AChE and MAO were assessed according to a colorimetric method described previously [29].

Measurement of AChE activity was performed based on the ability of AChE to hydrolyze acetylcholine iodide (ACTI) to thiocholine and acetic acid. In brief, 20 μL of the sample was mixed with a reaction mixture consisting of 200 μL of 0.1 mM sodium phosphate buffer (pH 8.0) and 10 μL of 0.2 M DTNB (5,5′-dithiobis(2-nitrobenzoic acid)). Then, the mixture was incubated at 25 °C for 5 min, and 10 μL of 15 mM acetylcholine thiochloride (ACTI) was added and incubated for 3 min. The yellow color substance generated was assessed at an optical density of 412 nm using a microplate reader, and the activity of AChE was expressed as nmol/min·mg.

In this study, the MAO assay was performed based on the ability of MAO to catalyze the oxidative deamination of biogenic amine substances such as tyramine. Briefly, a mixture comprising 50 μL of chromogenic solution, 200 μL of 500 μM of tyramine, and the sample aliquot at a volume of 50 μL was prepared, and the mixture was incubated at room temperature for 30 min. At the end of the incubation period, absorbance was determined at 490 nm. The activity of MAO was expressed as U/mg protein.

### 2.6. Determination of Lactobacillus spp. and Bifidobacterium spp. Density

The serial dilutions of feces in the concentration range of 10^−2^ to 10^−8^ were prepared by using phosphate buffer (pH 7.4) (1:9 *w*/*v*). A 100 μL aliquot measurement of each aforementioned serial dilution was inoculated in duplicate through surface spreading on De Man–Rogosa–Sharpe (MRS) agar plates (Himedia™ LACTOBACILLUS MRS AGAR) and Bifidobacterium agar (Himedia™) at 37 °C for 48 h in a W-Zip standing pouch by placing a GasPak™ anaerobic indicator (MGC, Mitsubishi, Tokyo, Japan) into the W-Zip bag in order to induce anaerobic conditions. Following this step, *Lactobacillus* spp. and *Bifidobacterium* spp. were separately isolated and counted according to the different morphologies observed on the culture plates and Gram-stained under a microscope. Data are expressed as the logarithm Colony-Forming Unit (CFU)/mL [30].

### 2.7. Statistical Analysis

Data of all measured parameters are presented as the mean ± S.E.M. The efficacy of an anthocyanin-enriched diet with a high DAQ-S but low DII was determined from the comparison of all health-related parameters before the intervention and at the end of the 5-day intervention period using a paired *t*-test. Statistical significance was considered at a *p*-value of <0.053.

## 3. Results

### 3.1. Study Population and General Characteristics

A total of 49 subjects were enrolled in this study. During the screening of eligible subjects, three individuals were excluded due to dyslipidemia. Thus, 12 male and 34 female volunteers met the inclusion criteria. The volunteers were randomly divided into two groups (N = 23/arm) consisting of the low-anthocyanin treatment group (those receiving an anthocyanin-enriched functional ingredient derived from black sticky rice at 2 g/day) and a high-anthocyanin treatment group (those receiving an anthocyanin-enriched functional ingredient derived from black sticky rice at 4 g/day). No subjects in either group withdrew from the study. In total, 4 males and 19 females consumed an anthocyanin-enriched diet at a dose of 2 g/day; in comparison, 8 males and 15 females consumed an anthocyanin-enriched diet at a dose of 4 g/day. No significant differences in age, vital signs, and body mass index (BMI) were observed between the two groups, as shown in Table 1.

### 3.2. Mental Health-Related Parameters

In this study, DASS-21 was used for assessing depression, anxiety, and stress and results were shown in Table 2. It was found that subjects who received anthocyanins-enrich diet at the doses of 2, and 4 g/day used in this study revealed the improvement in depression (*p*-value < 0.001, and 0.01, respectively; compared to before intervention), anxiety (*p*-value < 0.001, and 0.01, respectively; compared to before intervention), and stress (*p*-value < 0.001, and 0.01, respectively; compared to before intervention)

### 3.3. Changes in Neurotransmitters, Oxidative Stress Markers, and Inflammatory Mediators

A comparison of the results before and after the intervention (Table 3) indicates that subjects who consumed an anthocyanin-enriched diet for 5 days at a dose of 2 g/day with a low DII but high DAQ-S exhibited a slight elevation in MAO activity in serum (*p*-value < 0.05). No significant change in AChE activity was detected. The high dose of the anthocyanin-enriched diet (4 g/day) with a low DII but high DAQ-S also failed to yield significant differences in AChE or MAO.

The current data revealed that subjects who consumed anthocyanins-enriched diet at the doses of 2, and 4 g/day for 5 days significantly decreased IL-6 (*p*-value < 0.05 all; compared between before and after a 5-day intervention period, and 8-OHdG (*p*-value < 0.05, and 0.01, respectively; compared between before and after a 5-day intervention period). No significant changes in MDA and Nrf2 were observed in any groups as shown in Table 4.

### 3.4. Blood Chemistry Values Assessment

Since the consumption of a diet containing anthocyanin-enriched powder represents a novel method, safety-related parameters were also determined to ensure the efficacy of this method without inducing toxicity. It was found that volunteers who consumed an anthocyanin-enriched diet at doses of 2 and 4 g/day with a low DII but high DAQ-S demonstrated decreased bicarbonate (*p*-value < 0.01 and 0.001, comparing pre- and post-intervention), total protein (*p*-value < 0.01 for all participants, comparing pre- and post-intervention), and globulin levels (*p*-value < 0.001 for all participants, comparing pre- and post-intervention). The high-dose treatment also increased albumin levels but decreased fasting blood sugar (*p*-value < 0.05 for all participants, comparing pre- and post-intervention); in comparison, the low-dose treatment decreased potassium levels (*p*-value < 0.05, comparing pre- and post-intervention). No other changes were observed, as shown in Table 5. Despite the significant changes in the aforementioned parameters, all data were within a normal range. Therefore, the presented data do not reflect toxicity.

### 3.5. Changes in Lactobacillus spp. and Bifidobacterium spp. Density

A fecal analysis was also performed to determine the amount of *Lactobacillus* spp. and *Bifidobacterium* spp. in the volunteers’ feces. The present results demonstrate that there was no significant difference in both parameters, as shown in Table 6.

## 4. Discussion

The results of the present study demonstrate that the consumption of a diet with a low DII but high DAQ-S that contains anthocyanin-enriched powder from black sticky rice at the doses of 2 and 4 g/day significantly improves symptoms related to anxiety and depression, coupled with a reduction in 8-OHdG and IL-6 levels, in adult volunteers. Elevation in serum MAO activity was also observed in the subjects who consumed this diet at the dose of 2 g/day. In addition, the clinical chemistry values revealed no toxicity in any of the organs.

Imbalances in neurotransmitters such as gamma-aminobutyric acid (GABA) and the monoamines dopamine, norepinephrine, and serotonin play an important role in anxiety [31]. It has been reported that an increase in norepinephrine (NE), a crucial neuromodulator of arousal and vigilance, is strongly associated with anxiety [32]. Our data demonstrate an increase in MAO, an enzyme principally responsible for the degradation of amine neurotransmitters such as norepinephrine, epinephrine, serotonin, and dopamine [33], in subjects who consumed food containing an anthocyanin-enriched functional ingredient from black sticky rice at a dose of 2 g/day. These findings suggest a decrease in NE in this group of volunteers, resulting in an improvement in anxiety. Although an increase in MAO also increases the levels of other monoamine neurotransmitters, the main neurotransmitter that changes due to stress exposure is NE, which is released following sympathetic nervous system activation [34,35]. Therefore, the influence of MAO on NE in stressed volunteers appears to be greater than on other neurotransmitters because a high NE concentration, which serves as the substrate of MAO, is present during stress. However, a higher dose of the functional ingredient from black sticky rice (4 g/day) failed to exhibit a modulatory effect on MAO. One possible explanation may be due to the masking effect of other ingredients present in the black sticky rice extract. In this study, the functional ingredient was not pure anthocyanin but rather the crude extract of black sticky rice. As such, many other ingredients, in addition to anthocyanin, were also present in the extract. When the concentration of the functional ingredient increased, the concentration of other, non-active ingredients also increased, which could lead to masking the effect of the active ingredient. Due to the masking effect of the other ingredients, the higher dose of the functional ingredient from black sticky rice (4 g/day) did not result in a significant reduction in MAO induced by the possible active ingredient. In light of the above information, the improvement in anxiety and stress response observed in subjects who consumed an anthocyanin-enriched functional ingredient from black sticky rice at a dose of 2 g/day may partly occur due to an increase in MAO. However, this parameter may not be involved in the improvement in depression observed in this group.

In this study, stress-related symptoms were also assessed using the DASS-21. Based on the results of this test, these symptoms are related to norepinephrine overactivity [36]. The results of the DASS-21 demonstrate that the improvement in stress in subjects who consumed foods containing the functional ingredient from black sticky rice at the doses of 2 and 4 g/day may be partly associated with the increase in MAO, which, in turn, decreases the NE levels present. The subjects who consumed food containing the functional ingredient from black sticky rice at a dose of 4 g/day also showed an increase in MAO but with a loss of significance. In addition, NE activity depends on not only the inactivation process but also the synthesis of neurotransmitters. Therefore, the results may also indicate a decrease in NE synthesis. Unfortunately, NE synthesis was not analyzed, which represents a limitation of the study and necessitates further exploration to confirm this hypothesis.

Psychological stress exposure stimulates the sympathetic nervous system, resulting in an increase in norepinephrine-induced oxidative stress [37,38,39,40] through auto-oxidation [41]. As a result, oxidative stress induces the oxidation of the major biological constituents of DNA, lipids, and proteins, resulting in an increase in oxidative stress in the body, as evidenced by higher levels of 8-OHdG and MDA in serum and urine [42]. Therefore, the serum level of the aforementioned parameters can be used as an indicator of oxidative stress status [43].

This increase in oxidative stress induced by psychological stress can directly activate the conversion of tryptophan, a precursor for serotonin synthesis, to kynurenine. This change leads to a reduction in serotonin levels but induces the accumulation of kynurenine, which can be metabolized further to pro-oxidant compounds, such as 3-hydroxykynurenine and quinolinic acid, associated with the pathogenesis of depression [4]. The activation of this system can also occur indirectly through the increase in inflammation caused by oxidative stress. Both serotonin deficiency and the accumulation of kynurenine metabolites such as 3-hydroxykynurenine and quinolinic acid are believed to be involved in the development of depression [44]. In addition, serotonin deficiency, particularly in prefrontal–cortical circuits, also plays a role in the development of anxiety [18]. The above evidence suggests that the reduction in 8-OHdG and IL-6 levels, which reflects the decrease in oxidative stress and inflammation, may play an important role in reducing depression, anxiety, and stress in volunteers who consumed food containing an anthocyanin-enriched functional ingredient from black sticky rice at both doses used in this study.

Oxidative stress also induces neurodegeneration in various bodily systems. The neurodegeneration of the GABAergic system in the basolateral nucleus of the amygdala can induce behavioral hyperexcitability, such as increased anxiety and depression, and emotional dysregulation [45]. Therefore, the reduction in oxidative stress and inflammation observed in the volunteers who consumed diets containing the functional ingredient from black sticky rice at doses of 2 and 4 g/day may improve GABAergic function, resulting in an improvement in anxiety-, depression-, and stress-related symptoms in this study. However, GABAergic function was not assessed in this study, which is a limitation of the study and requires further investigation to provide a better and more in-depth understanding of the mechanism.

Owing to the effect of anthocyanins on the modulation of the gut microbiota, which in turn stimulates the gut–brain axis, inducing the regulation of central nervous system function [22], we also examined the alteration in microbiota, particularly *Lactobacillus* and *Bifidobacterium* spp., in the feces of volunteers who consumed diets containing the functional ingredient from black sticky rice at doses of 2 and 4 g/day. However, no significant changes in the aforementioned parameters were observed in this study. This may be because the duration of the study was too short to determine any changes in the observed parameters. Thus, the role of the functional ingredient from black sticky rice on the modulation of the gut microbiota and the stimulation of the gut–brain axis requires further study over a longer period.

It has been reported that many factors such as food [46], physical activity, exercise [47], sleep, and the menstrual cycle [48] can also exert specific influences on the alteration in oxidative stress and inflammation. The quantity of various types of food and the physical activity levels of the subjects in this study were assessed, and the obtained data are shown in Supplementary Material S2 (Table S1) and Supplementary Material S3 (Table S2). The results indicate no close association between alterations in oxidative stress and inflammation and changes in food intake and physical activity. Therefore, the observed changes in oxidative stress and inflammation in this study did not occur due to changes in food intake and physical activity. We also examined sleep problems at each visit and none of the volunteers reported such issues during the study period. All the female subjects included in this study had regular menstrual cycles, and to avoid a confounding impact induced by different phases of the menstrual cycle, during the 5-day study period, they all remained in the same phase of their menstrual cycle. Each phase of the menstrual cycle exerts a different influence on oxidative stress and inflammation. It has been demonstrated that the luteal phase of the menstrual cycle is associated with higher levels of oxidative stress than the follicular phase because the luteal phase is dominated by the production of progesterone, whereas the follicular phase is dominated by estradiol, which exhibits antioxidant effects [49,50,51]. Due to the conditions in this study, differences in sleep and the menstrual cycle were less likely to induce confounding impacts on oxidative stress and inflammation changes.

The functional ingredient from black sticky rice used in this study is rich in anthocyanins, such as cyanidin-3-glucoside, cyanidin-3-rutinoside, and peonidin, which exhibit antioxidant and anti-inflammatory effects [52]. Cyanidin compounds also exhibit antidepressive-like activity [53] and anxiolytic effects [54]. Based on these findings, we suggest that the mental health benefits observed in this study may be associated with anthocyanins, particularly cyanidin compounds.

This study presents an interesting application of functional ingredients in diet. In addition, the results of this pilot study demonstrate mental health benefits with the consumption of this diet for 5 days. The strength of this study is the assessment of mental well-being with well-validated assessment tools such as the DASS-21, which is widely accepted and used for assessment in the Department of Psychiatry at our institution. However, this study has several limitations; namely, the assessment of the transmitter turnover rate focused only on inactivation and not synthesis, the sample size was small due to the pilot study, and the duration of the study was not sufficient to detect any alteration in the modulation of gut microbiota and the gut–brain axis.

## 5. Conclusions

In conclusion, this study is the first that clearly demonstrates the mental health benefits associated with the consumption of food containing an anthocyanin-enriched functional ingredient from black sticky rice with a low DII and high DAQ-S over a 5-day period. The possible mechanism may partly be associated with the reduction in oxidative stress and inflammation. An increase in MAO, which in turn improves noradrenergic function, also plays a role. However, randomized double-blind, placebo-controlled studies with a larger sample size are required to confirm the beneficial effects of the application of the functional ingredient from black sticky rice.

## Figures and Tables

**Table 1:** General characteristics of subjects who received a diet containing a black sticky rice-derived functional ingredient at doses of 2 and 4 g per day.

Parameters	Anthocyanins-Enriched Diet (2 g/day)		Anthocyanins-Enriched Diet (4 g/day)	
	Baseline (*n* = 23)	5-day (*n* = 23)	Baseline (*n* = 23)	5-day (*n* = 23)
Age (year)	44.91 ± 1.66	44.91 ± 1.66 (*p* = 1.000)	39.70 ± 1.89	39.70 ± 1.89 (*p* = 1.000)
Gender (Male/Female)	4/19	4/19	8/15	8/15
Body Temperature (°C)	36.58 ± 0.04	36.58 ± 0.04 (*p* = 1.000)	36.60 ± 0.02	36.56 ± 0.03 (*p* = 0.584)
Heart rate (beats/min)	72.17 ± 1.99	72.17 ± 1.99 (*p* = 1.000)	74.48 ± 2.39	75.04 ± 1.08 (*p* = 0.851)
Respiratory rate (breaths/min)	17.00 ± 0.09	17.00 ± 0.09 (*p* = 1.000)	17.04 ± 0.10	16.87 ± 0.10 (*p* = 0.211)
Systolic BP (mmHg)	111.57 ± 2.44	111.57 ± 2.44 (*p* = 1.000)	116.09 ± 2.65	112.39 ± 2.49 (*p* = 0.314)
Diastolic BP (mmHg)	70.57 ± 1.91	70.57 ± 1.91 (*p* = 1.000)	74.22 ± 2.13	72.04 ± 1.66 (*p* = 0.425)
Body Weight (kg)	57.15 ± 1.67	57.15 ± 1.67 (*p* = 1.000)	59.29 ± 1.63	59.67 ± 1.70 (*p* = 0.873)
Body Height (cm)	157.70 ± 1.54	157.70 ± 1.54 (*p* = 1.000)	161.61 ± 1.54	161.61 ± 1.54 (*p* = 1.000)
BMI (kg/m^2^)	22.89 ± 0.38	22.89 ± 0.38 (*p* = 1.000)	22.68 ± 0.49	22.84 ± 0.54 (*p* = 0.792)

**Table 2:** The Depression Anxiety Stress Scale-21 (DASS-21) scores of adult volunteers who consumed diet containing anthocyanin-enrich functional ingredient from black sticky rice (N = 23/arm). Data were presented as mean ± S.E.M. **, *** *p*-value < 0.01, and 0.001, respectively; compared between before and after a 5-day study period.

Parameters	Anthocyanins-Enriched Diet (2 g/day)		Anthocyanins-Enriched Diet (4 g/day)	
	Baseline (*n* = 23)	5-day (*n* = 23)	Baseline (*n* = 23)	5-day (*n* = 23)
Depression	2.08 ± 0.44	0.56 ± 0.18 (*p* < 0.001) ***	4.00 ± 0.49	2.26 ± 0.59 (*p* < 0.001) **
Anxiety	2.13 ± 0.29	0.69 ± 0.18 (*p* < 0.001) ***	3.04 ± 2.87	2.13 ± 1.39 (*p* < 0.001) **
Stress	3.08 ± 0.47	1.52 ± 0.45 (*p* < 0.001) **	6.52 ± 0.62	3.60 ± 0.69 (*p* < 0.001) **

**Table 3:** Acetylcholinesterase and monoamine oxidase activities in serum of adulthood volunteers who consumed diet containing anthocyanins-enriched functional ingredient from black stick rice at doses of 2 and 4 g/day. (N = 23/arm). Data were presented as mean ± S.E.M. * *p*-value < 0.05; compared between before and after a 5-day study period.

Parameters	Anthocyanins-Enriched Diet (2 g/day)		Anthocyanins-Enriched Diet (4 g/day)	
	Baseline (*n* = 23)	5-day (*n* = 23)	Baseline (*n* = 23)	5-day (*n* = 23)
MAO activity (μmol/mg protein)	0.31 ± 0.01	0.36 ± 0.02 * (*p* = 0.027)	0.29 ± 0.01	0.33 ± 0.01 (*p* = 0.097)
AChE activity (nmol/mg protein)	0.12 ± 0.00	0.11 ± 0.01 (*p* = 0.067)	0.12 ± 0.00	0.11 ± 0.00 (*p* = 0.341)

**Table 4:** Inflammatory mediator (IL6), and oxidative stress biomarkers including 8-OHdG, MDA, IL-6 and Nrf2 in serum of adulthood volunteers who consumed diet containing anthocyanins-enriched functional ingredient from black sticky rice at doses of 2 and 4 g/day (N = 23/arm). Data were presented as mean ± S.E.M. *,** *p*-value < 0.05, and 0.01, respectively; compared between before and after a 5-day study period.

Parameters	Anthocyanins-Enriched Diet (2 g/day)		Anthocyanins-Enriched Diet (4 g/day)	
	Baseline (*n* = 23)	5-day (*n* = 23)	Baseline (*n* = 23)	5-day (*n* = 23)
MDA (µmol/L)	3.75 ± 0.57	2.60 ± 0.14 (*p* = 0.055)	3.87 ± 0.47	2.89 ± 0.18 (*p* = 0.070)
8-OHdG (ng/mL)	169.54 ± 15.95	110.25 ± 16.30 * (*p* = 0.013)	191.11 ± 15.94	110.58 ± 17.15 ** (*p* = 0.001)
IL-6 (pg/mL)	3.90 ± 0.50	2.79 ± 0.28 * (*p* = 0.033)	3.74 ± 0.33	2.56 ± 0.34 * (*p* = 0.004)
Nrf2 (pg/mL)	58.20 ± 12.34	44.03 ± 8.50 (*p* = 0.350)	135.64 ± 27.51	113.29 ± 33.14 (*p* = 0.250)

**Table 5:** Blood chemistry values in a serum of adulthood volunteers who consumed diet containing anthocyanins-enriched functional ingredient from black sticky rice at doses of 2 and 4 g/day. (N = 23/arm) *, **, *** *p*-value < 0.05, 0.01, and 0.001, respectively; compared between before and after a 5-day study period.

Parameters	References	Anthocyanins-Enriched Diet (2 g/day)		Anthocyanins-Enriched Diet (4 g/day)	
		Baseline (*n* = 23)	5-day (*n* = 23)	Baseline (*n* = 23)	5-day (*n* = 23)
BUN (Blood Urea itrogen)	5.8–19.1 mg/dL	10.38 ± 0.59	10.84 ± 0.43 (*p* = 0.526)	10.63 ± 0.54	12.35 ± 0.67 (*p* = 0.065)
Creatinine	0.5–1.5 mEq/L	0.84 ± 0.03	0.82 ± 0.02 (*p* = 0.921)	0.82 ± 0.02	0.86 ± 0.03 (*p* = 0.312)
Sodium	130–147 mEq/L	138.35 ± 0.28	137.87 ± 0.28 (*p* = 0.091)	137.91 ± 0.27	137.74 ± 0.29 (*p* = 0.801)
Potassium	3.4–4.7 mEq/L	4.45 ± 0.08	4.22 ± 0.07 * (*p* = 0.040)	4.45 ± 0.06	4.44 ± 0.06 (*p* = 0.918)
Bicarbonate	20.6–28.3 mEq/L	22.64 ± 0.33	20.86 ± 0.42 ** (*p* = 0.002)	22.92 ± 0.32	21.27 ± 0.26 *** (*p* ≤ 0.001)
Chloride	96–107 mEq/L	101.52 ± 0.26	102.09 ± 0.46 (*p* = 0.309)	100.78 ± 0.35	100.74 ± 0.33 (*p* = 0.929)
Total Protein	6.5–8.8 g/dL	7.80 ± 0.07	7.47 ± 0.06 ** (*p* = 0.001)	7.97 ± 0.08	7.59 ± 0.08 ** (*p* = 0.002)
Albumin	3.8–5.4 g/dL	4.24 ± 0.05	4.35 ± 0.05 (*p* = 0.141)	4.31 ± 0.05	4.46 ± 0.04 * (*p* = 0.026)
Globulin	2.6–3.4 g/dL	3.55 ± 0.07	3.11 ± 0.06 *** (*p* ≤ 0.001)	3.67 ± 0.07	3.14 ± 0.07 *** (*p* ≤ 0.001)
Total Bilirubin	0.3–1.5 mg/dL	0.49 ± 0.07	0.53 ± 0.06 (*p* = 0.126)	0.44 ± 0.04	0.47 ± 0.04 (*p* = 0.338)
Direct Bilirubin	0.0–0.5 mg/dL	0.20 ± 0.02	0.20 ± 0.02 (*p* = 0.597)	0.19 ± 0.02	0.18 ± 0.01 (*p* = 0.822)
ALT	4–36 U/L	15.39 ± 1.68	18.22 ± 2.76 (*p* = 0.367)	22.26 ± 2.81	22.26 ± 2.14 (*p* = 0.488)
AST	12–32 U/L	19.13 ± 1.02	21.52 ± 1.39 (*p* = 0.173)	23.43 ± 1.84	23.43 ± 1.24 (*p* = 0.552)
ALP	42–121 U/L	73.61 ± 3.53	71.35 ± 3.09 (*p* = 0.632)	69.13 ± 3.05	69.35 ± 3.44 (*p* = 0.963)
Homocysteine	0–15 U/L	11.04 ± 0.62	11.83 ± 0.53 (*p* = 0.198)	10.52 ± 0.53	11.39 ± 0.62 (*p* = 0.236)
Cortisol in Blood	6.2–19.4 μg/dL	7.95 ± 0.52	8.80 ± 0.67 (*p* = 0.392)	10.02 ± 0.71	9.89 ± 0.98 (*p* = 0.717)
Cholesterol	127–262 mg/dL	202.26 ± 5.10	201.43 ± 6.19 (*p* = 0.918)	215.96 ± 6.56	207.13 ± 6.71 (*p* = 0.352)
Triglycerides	10–200 mg/dL	115.17 ± 161.75	103.96 ± 13.73 (*p* = 0.607)	116.00 ± 9.16	100.43 ± 8.40 (*p* = 0.235)
HDL-Chol	>35 mg/dL	58.96 ± 2.98	60.13 ± 3.08 (*p* = 0.785)	60.70 ± 3.74	62.13 ± 3.51 (*p* = 0.860)
LDL-Chol (DIRECT)	10–150 mg/dL	124.83 ± 4.53	129.48 ± 6.35 (*p* = 0.554)	134.91 ± 6.10	132.83 ± 6.64 (*p* = 0.818)
Atherogenic index	<0.11	0.25 ± 0.06	0.20 ± 0.06 (*p* = 0.652)	0.24 ± 0.05	0.19 ± 0.05 (*p* = 0.311)
AI	-	2.62 ± 0.21	2.55 ± 0.23 (*p* = 0.684)	2.79 ± 0.20	2.52 ± 0.18 (*p* = 0.326)
FBS	70–110 mg/dL	101.78 ± 1.87	101.55 ± 1.92 (*p* = 0.930)	109.09 ± 2.20	100.04 ± 2.86 * (*p* = 0.016)
Insulin	2.6–24.9 μU/mL	7.06 ± 0.74	7.38 ± 0.92 (*p* = 0.982)	8.15 ± 1.13	8.04 ± 1.17 (*p* = 0.878)
HOMA-IR	-	1.80 ± 0.21	1.85 ± 0.24 (*p* = 0.982)	2.19 ± 0.32	2.05 ± 0.33 (*p* = 0.503)

**Table 6:** Amount of *Lactobacillus* and *Bifidobacterium* spp. in feces of adulthood volunteers who consumed dirt containing anthocyanin-enriched functional ingredient from black sticky rice. at doses of 2 and 4 g/day. (N = 23/arm).

Parameters	Anthocyanins-Enriched Diet (2 g/day)		Anthocyanins-Enriched Diet (4 g/day)	
	Baseline (*n* = 23)	5-day (*n* = 23)	Baseline *(n* = 23)	5-day (*n* = 23)
Lactic acid producing bacteria (Log CFU)/mL)	7.22 ± 0.40	8.15 ± 0.19 (*p* = 0.103)	6.91 ± 0.44	7.53 ± 0.37 (*p* = 0.217)
*Lactobacillus* spp. (Log CFU)/mL)	5.67 ± 0.36	6.12 ± 0.41 (*p* = 0.264)	5.49 ± 0.49	5.98 ± 0.44 (*p* = 0.438)
*Bifidobacterium* spp. (Log CFU)/mL)	5.89 ± 0.46	6.15 ± 0.44 (*p* = 0.668)	4.72 ± 0.34	5.42 ± 0.40 (*p* = 0.193)

## Data Availability

The data presented in this study are available on request from the corresponding author.

## Supplementary Materials

The following supporting information can be downloaded at: https://www.mdpi.com/article/10.3390/foods13233884/s1. Supplementary Material S1 is the fingerprint chromatogram of anthocyanin-enrich functional ingredient, S2 is the food consumption of the volunteers, and S3 is the physical activity of the volunteers.
