# Supplementary material for: A Black Sticky Rice-Derived Functional Ingredient Improves Anxiety, Depression, and Stress Perception in Adult Volunteers
Source: Foods. 2024 Dec 1;13(23):3884. doi: 10.3390/foods13233884 (PMC11640055; doi:10.3390/foods13233884)
Supplement: Supplementary file 1 [file foods-13-03884-s001.zip › S1 HPLC anthocyanin enrich functional ingredient.pdf]

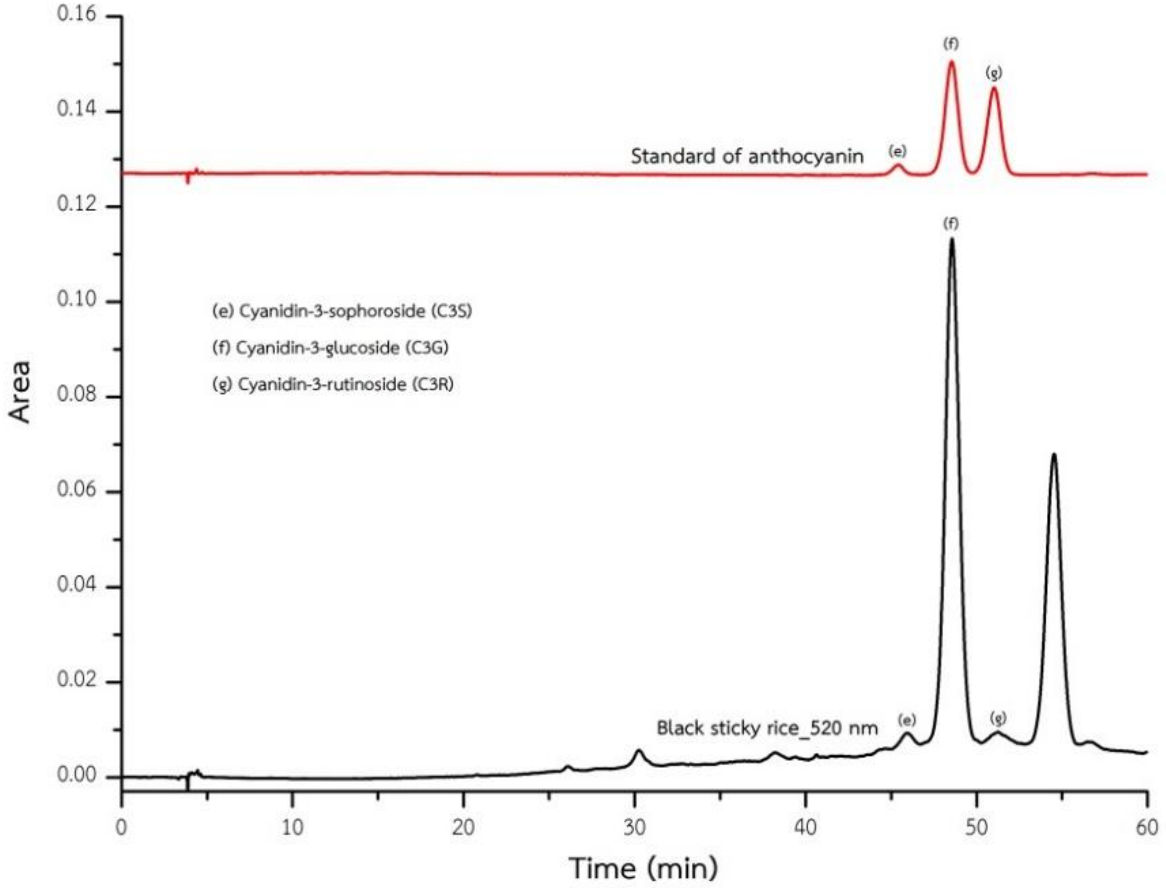

Figure S1 Chromatogram of anthocyanin-enrich functional ingredient at a concentration of 20μg/ml
