# Supplementary material for: A Black Sticky Rice-Derived Functional Ingredient Improves Anxiety, Depression, and Stress Perception in Adult Volunteers
Source: Foods. 2024 Dec 1;13(23):3884. doi: 10.3390/foods13233884 (PMC11640055; doi:10.3390/foods13233884)
Supplement: Supplementary file 1 [file foods-13-03884-s001.zip › S2 Table FFQ.pdf]

Table S1 Food frequency questionnaire of adulthood volunteers who consumed anthocyanins-enriched diet at doses of 2 and 4 g/day with low DII but high DAQ-S. (N=23/arm). Data were presented as mean±S.E.M. *p-value<0.05; compared between before and after a 5-day study period.

| **Parameters** | **Anthocyanins-enriched diet (2 g/day)** | | **Anthocyanins-enriched diet (4 g/day)** | |
|---|---|---|---|---|
| | **Baseline (N=23)** | **5-day (N=23)** | **Baseline (N=23)** | **5-day (N=23)** |
| Rice cooked (scoop) | 22.50±1.81 | 26.85±1.92 (p=0.107) | 28.57±2.97 | 27.85±2.42 (p=0.956) |
| Meat (piece) | 33.37±6.11 | 30.17±4.66 (p=0.843) | 44.52±6.78 | 57.22±7.65 (p=0.187) |
| Egg | 11.87±1.53 | 12.04±1.82 (p=0.942) | 8.13±1.60 | 7.91±1.61 (p=0.826) |
| Milk (glass/box) | 6.70±1.27 | 5.83±1.07 (p=0.921) | 2.35±0.37 | 2.70±0.76 (p=0.972) |
| Vegetables (cup) | 8.02±1.65 | 10.91±1.77 (p=0.244) | 9.96±1.64 | 13.20±1.84 (p=0.193) |
| Fruit (cup) | 8.26±1.63 | 9.70±1.68 (p=0.465) | 6.48±1.67 | 10.26±1.72 *(p=0.025) |
