# Supplementary material for: A Black Sticky Rice-Derived Functional Ingredient Improves Anxiety, Depression, and Stress Perception in Adult Volunteers
Source: Foods. 2024 Dec 1;13(23):3884. doi: 10.3390/foods13233884 (PMC11640055; doi:10.3390/foods13233884)
Supplement: Supplementary file 1 [file foods-13-03884-s001.zip › S3 Table S2 Physical activity.pdf]

Table S2 Physical activity record of volunteers who received a set menu containing anthocyanin-enrich functional ingredient from black sticky rice at the doses of 2 and 4 grams per day, before and after consuming the product for 5 days (Mean±SEM).

Parameters		Subjects who consumed anthocyanin-rich functional ingredient from black sticky rice 2 g/day		Subjects who consumed anthocyanin-rich functional ingredient from black sticky rice 4 g/day	
		Before intervention (N=46)	After a 5-day consumption period (N=46)	Before intervention (N=45)	After a 5-day consumption period (N=45)
Strenuous Activities	Fast running	0.46±0.19	0.83±0.26 (p=0.206)	0.64±0.24	0.98±0.29 (p=0.516)
	Heavy Lifting	1.46±0.41	1.91±0.44 (p=0.625)	1.36±0.37	1.62±0.42 (p=0.708)
	Exercise (minutes.)	70.13±17.63	76.43±18.97 (p=0.990)	78.22±16.64	90.44±24.71 (p=0.839)
Moderate Strength Activities	Cycling on flat ground	1.09±0.35	0.78±0.30 (p=0.531)	1.13±0.35	1.24±0.37 (p=0.668)
	Light Lifting	1.67±0.43	1.74±0.43 (p=0.949)	1.18±0.37	1.44±0.40 (p=0.598)
Walking	Fast Walking	2.63±0.45	2.41±0.46 (p=0.737)	2.40±0.45	2.60±0.46 (p=0.577)
	Slow Walking	4.15±0.44	4.41±0.45 (p=0.674)	3.96±0.47	4.24±0.46 (p=0.557)
	Walking at home	4.65±0.41	4.46±0.43 (p=0.850)	4.13±0.46	4.56±0.46 (p=0.468)
	Walking to places	4.72±0.43	4.52±0.46 (p=0.736)	4.33±0.45	4.58±0.47 (p=0.627)
	Walking to Leisure	2.83±0.45	3.04±0.48 (p=0.787)	2.18±0.45	2.18±0.45 (p=0.921)
Sitting	Sit at the desk	3.24±0.44	3.76±0.48 (p=0.264)	3.51±0.46	3.38±0.47 (p=0.819)
	Sit and watch TV	3.65±0.42	4.13±0.43 (p=0.395)	4.13±0.44	4.07±0.44 (p=0.970)
	Sit and chat with friends	4.09±0.44	4.46±0.43 (p=0.544)	4.00±0.46	4.42±0.44 (p=0.485)
	Sit and relax	4.78±0.43	4.54±0.43 (p=0.710)	3.80±0.44	4.82±0.43 (p=0.066)
	Sit and read a book	0.63±0.24	0.83±0.28 (p=0.470)	1.07±0.33	0.98±0.32 (p=0.957)
Miscellaneous Activities		0.00±0.00	0.00±0.00	0.00±0.00	0.00±0.00
