# Supplementary material for: A Black Sticky Rice-Derived Functional Ingredient Improves Anxiety, Depression, and Stress Perception in Adult Volunteers
Source: Foods. 2024 Dec 1;13(23):3884. doi: 10.3390/foods13233884 (PMC11640055; doi:10.3390/foods13233884)
Supplement: Supplementary file 1 [file foods-13-03884-s001.zip › S4 A 5-day food menu.pdf]

	Menu	Amount/ Serving	Energy (Kcal)	Protein (g)	CHO (g)	Fat (g)	Sodium (mg)	Dietary Fiber(g)	DII	DAQS
Day-1										
Breakfast	Fish maw soup	350.00	419.28	3.39	32.62	15.61	1812.88	2.20	-3.115	3.115
	Jelly drink made from rice water mixed with functional ingredients	150.00	272.10	6.38	60.17	1.56	13.16	3.04	-2.32	2.32
Lunch	Sticky rice burger with Chicken Larb	150.00	340.45	7.35	53.65	6.69	638.55	2.89	-4.36	4.36
	Papaya salad with bamboo shoots	150.00	147.60	1.97	30.67	1.69	687.41	1.26	-0.93	0.93
	Oatmeal Cookies	30.00	233.72	34.17	29.65	11.94	149.66	1.05	-0.322	0.322
	Jackfruit	40.00	38.00	4.25	9.20	0.24	0.80	0.60	-0.4	0.4
Dinner	Rice	150.00	195.00	1.80	42.00	0.45	1.50	0.60	-1.5	1.5
	Spicy tuna salad	120.00	275.42	4.98	60.14	0.74	787.53	2.69	-1.01	1.01
	Liang soup with shrimp	250.00	132.24	2.46	15.71	1.37	1302.74	5.33	-1.125	1.125
	Watermelon	120.00	40.80	0.50	9.12	0.24	1.20	0.48	-1.2	1.2
Day-2										
Breakfast	Whole Wheat Sandwich with Crab Sticks	190.00	487.36	4.58	60.51	20.83	611.51	6.57	-3.6	3.6
	Two-Color Rice Pudding (Add functional ingredient)	100.00	243.44	7.48	50.93	5.15	31.14	1.28	-1.56	1.56
Lunch	Teriyaki Chicken Rice	250.00	384.68	4.59	32.75	15.95	1590.32	3.26	-3.96	3.96
	Miso Soup	200	19.3	0.1	0.8	1.94	151.06	0.14	-0.28	0.28
	Caramelized Banana	160.00	419.46	0.91	85.94	8.60	85.22	1.90	-3.7	3.7
Dinner	Rice	150.00	195.00	1.80	42.00	0.45	1.50	0.60	-1.5	1.5
	Chicken boiled with tamarind leaves	200.00	169.46	3.20	15.06	8.04	1004.12	1.56	-0.78	0.78
	Shrimp stir-fried with chili and salt	80.00	161.98	18.96	8.13	10.29	714.44	0.22	-1.04	1.04
Day-3										
3 เช้า	Anthocyanin-rich ball (add functional ingredient)+ Grilled pork with basil	150.00	404.48	10.20	50.85	11.94	739.44	1.82	-2.19	2.19

	Pineapple	120.00	60.00	0.42	15.60	0.12	1.20	1.68	-1.2	1.2
Lunch	Green curry fried rice with chicken wrapped in eggs	200.00	353.84	3.97	53.39	7.83	524.35	0.67	-0.311	0.311
	Spicy Dory fish salad	100.00	183.04	11.87	15.21	8.30	800.31	0.93	-1.2	1.2
	Young coconut pudding	80	124.14	3.00	10.79	8.61	332.20	0.01	-1.93	1.93
Dinner	Grilled Nile Tilapia Miang	300.00	348.68	3.32	32.70	12.65	1177.10	2.11	-2.9	2.9
	Inthanin dessert	120.00	175.40	1.99	22.80	8.10	71.38	2.19	-1.3	1.3
Day-4										
Breakfast	purple cream soup with ginger and coconut (Add functional ingredient)	200.00	316.14	3.05	36.74	13.75	175.23	2.80	-2.59	2.59
	Pudding	100.00	243.44	7.48	50.93	5.15	31.14	1.28	-1.56	1.56
Lunch	Ginger Fried Rice	200.00	394.26	2.02	72.57	7.40	422.43	0.82	-2.05	2.05
	Fried Dolly Fish with Ginger Sauce	100.00	141.67	6.51	20.56	3.74	605.21	0.99	-0.57	0.57
	Clear Soup and Chicken Terrine, Chinese Dates, Wood Ear Mushrooms	200.00	73.13	1.39	6.36	2.86	231.97	0.22	-1.1	1.1
Dinner	Rice	150.00	195.00	1.80	42.00	0.45	1.50	0.60	-1.5	1.5
	Tom Klong Gumbucha Coffee Grounds with Stewed Pork	250.00	90.14	1.15	13.44	2.03	280.14	0.99	-0.15	0.15
	Fried Herb-Marinated Shrimp	100.00	140.70	12.08	13.85	5.69	667.12	1.55	-0.99	0.99
Day-5										
Breakfast	Dry rice porridge with minced pork and mushrooms	350.00	762.14	3.68	79.83	28.00	633.15	3.09	-2.94	2.94
	Ripen Papaya	100.00	43.00	0.50	11.00	0.30	8.00	1.70	-1	1
Lunch	spicy purple color Thai sushi (Add functional ingredient)	100.00	402.26	17.29	66.28	8.41	1105.06	5.45	-3.95	3.95
	Grilled Chicken	100.00	199.97	19.51	5.40	11.68	508.14	0.10	-0.99	0.99
	Kaeng Om Fish Curry Roll with Collagen	250.00	47.62	0.92	5.04	0.72	23.56	0.49	-0.96	0.96
	Somchun	150.00	163.10	0.24	41.94	0.14	3.61	0.65	-1.17	1.17

Dinner	Grilled Chicken Breast Steak + Vegetable Salad	400.00	765.03	2.77	34.68	50.65	1791.06	1.22	-3.23	3.23
	Mulberry Cheesecake with Three Flavors	100.00	294.81	4.15	26.43	19.31	268.48	0.44	-1.3	1.3
